# Computer-Aided Assessment of Three-Dimensional Standard Bone Morphology of the Distal Radius

**DOI:** 10.3390/diagnostics12123212

**Published:** 2022-12-17

**Authors:** Akira Ikumi, Yuichi Yoshii, Yusuke Eda, Tomoo Ishii

**Affiliations:** 1 Department of Orthopaedic Surgery, Tsukuba University Hospital, Tsukuba, Ibaraki 305-8576, Japan; 2 Department of Orthopaedic Surgery, Tokyo Medical University Ibaraki Medical Center, Ami, Ibaraki 300-0395, Japan; 3 Department of Orthopaedic Surgery, Mito Kyodo General Hospital, Mito, Ibaraki 310-0015, Japan

**Keywords:** distal radius, computed tomography, three dimensions, osteosynthesis, computer-aided diagnosis

## Abstract

The present study attempted to define the three-dimensional (3D) locations of reference points and standard measures of the distal radius of a normal wrist joint. One hundred wrists from 50 males and 50 females who matched the age distribution (19–95 years old, mean: 56.0 years old) were evaluated. Computed tomography (CT) images of normal wrist joints acquired for comparison with the affected side were used. The absence of a previous history and complaints in the unaffected wrist was confirmed in an interview and with medical records. Three-dimensional images of the distal radius were reconstructed using the data obtained from CT scans. The site at which the major axis of the radial diaphysis contacted the distal radius joint surface was defined as the origin. The 3D coordinates of reference points for the radial styloid process (1), sigmoid notch volar edge (2), and sigmoid notch dorsal edge (3) as well as the barycenter for the joint surface and joint surface area were evaluated. A slope of the line connecting coordinates 1–2 in the coronal plane was evaluated as the 3D radial inclination (3DRI) and that connecting coordinates 2–3 in the sagittal plane as the 3D palmar tilt (3DPT). Each measurement value was compared between males and females. The positions of each reference point from the origin were as follows: (1) 14.2 ± 1.3/12.6 ± 1.1 mm for the distal-palmar-radial position; (2) 19.3 ± 1.3/16.9 ± 1.3 mm for the proximal-palmar-ulnar position; (3) 15.6 ± 1.4/14.1 ± 0.9 mm for the proximal-dorsal-ulnar position; and (barycenter) 4.1 ± 0.7/3.7 ± 0.7 mm for the proximal-volar-ulnar position for males and females, respectively. The areas of the radius articular surface were 429.0 ± 67.9/347.6 ± 44.6 mm^2^ for males and females, respectively. The 3DRI and 3DPT were 24.2 ± 4.0/25.7 ± 3.1° and 10.9 ± 5.1/13.2 ± 4.4° for males and females, respectively. Significant differences were observed in all measurement values between males and females (*p* < 0.01). The reference points and measured values obtained in the present study will serve as criteria for identifying the dislocation direction and reduction conditions of distal radius fractures in 3D images.

## 1. Introduction

A three-dimensional (3D) assessment of the distal radius based on computer tomography (CT) images has become commonplace in the treatment of distal radius fractures [1,2,3]. The 3D-CT evaluations revealed that differences in fracture patterns were dependent on displacement directions and that intra-articular fractures were more likely to occur between ligament attachments [4]. Through these evaluations, the patterns of distal radius fractures may be analyzed three-dimensionally based on anatomical landmarks. Visualizations of the fracture line and displacement direction using 3D images are useful for the formulation of treatment strategies. In plain radiographs, radial inclination (RI) and palmar tilt (PT) are usually used as the defined measurement index of distal radius. In the treatment of distal radius fractures, these parameters; healthy side or previously defined normal values are used as indices of fracture reduction. However, as these parameters are measured as a 3D structure in 2D view, it has a potential risk of measurement errors and individual differences in normal values which may affect the treatment outcome. On the other hand, there are no defined measurement indices and standard values of the distal radius in 3D evaluation.

Computer-assisted technology has recently been applied to analyses of displacement patterns, the mechanisms of injuries, virtual planning, and simulations of osteosynthesis in distal radius fractures [2,5]. 3D quantitative evaluation methods for the amount of dislocation and analyses of stress distribution with the finite element method have contributed to a more detailed understanding of the pathophysiology of distal radius fractures. Furthermore, advances have been achieved in computer-assisted surgery for distal radius fractures [6,7,8]. Some 3D-printed models, preoperative simulations, and navigation for osteosynthesis have been reported. We previously evaluated the reproducibility of 3D preoperative planning for distal radius fractures [9]. The findings obtained revealed that 3D preoperative planning for osteosynthesis in distal radius fractures was reproducible with an error of approximately 2 mm for anatomical reference points and the correlations of reduction shapes were moderate. However, there are currently few criteria for the 3D standard morphology of the distal radius. To date, studies on the 3D morphology of the distal radius have been performed using local morphological and morphometric evaluations based on CT images [10,11]. Although the methods used in a 3D analysis of distal radius fractures have undergone extensive development, the reference points that serve as criteria for reductions in the treatment of fractures have not yet been defined. To elucidate the 3D positional relationship of reference points in the distal radius, which will help surgeons to avoid suboptimal reductions and related complications, we defined the three edges of the distal radius articular surface as distinct reference points. The present study attempted to define the standard measures of the distal radius for each gender which was calculated by defined 3D reference points in normal wrist joints. We hypothesized differences in the 3D standard bone shape of the distal radius between males and females.

## 2. Materials and Methods

The protocol of this retrospective case control study (level of evidence III) was approved by the Institutional Review Board. The radiographic database was accessed to identify cases that underwent a CT scan of the normal wrist. Using an image database, we evaluated CT images of unaffected wrists taken for comparison with the affected side. The absence of a previous history and complaints in the unaffected wrist was confirmed in an interview and with medical records. CT images of 100 wrists from 50 males and 50 females who matched the age distribution (19–95 years old, mean: 56.1 years old for males; 18–93 years old, mean: 58.8 years old for females) were evaluated. Patients were excluded if they had a previous history of traumatic arm injuries or were younger than 18 years. CT imaging conditions were as follows: a tube setting of 120 kV and 100 mAS; a section thickness of 1–1.5 mm; and a pixel size of 0.3 × 0.3 mm (Sensation Cardiac, Siemens, Berlin, Germany). CT images were taken from the metacarpal bone level to approximately 13 cm proximal to the radius joint surface.

### 2.1. 3D Bone Morphology Analysis

**Figure 1:**
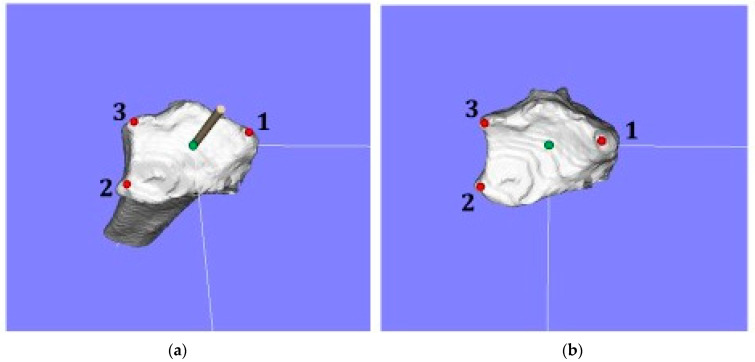
Three reference points on 3D images. (**a**) The oblique view from the distal ulnar side and (**b**) the axial view. Red dots indicate the three reference points: (1) the radial styloid process; (2) sigmoid notch volar edge; and (3) sigmoid notch dorsal edge. The long axis of the radius was marked by the green dots and bar.

**Figure 2:**
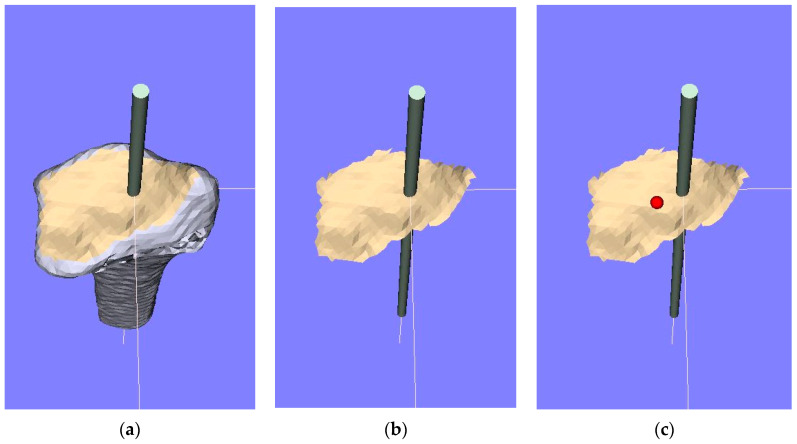
Articular surface and the position of barycentric coordinates. (**a**) The articular surface of the 3D model was identified with the bony prominence of the distal radius, (**b**) the articular surface was extracted, and (**c**) the surface area and position of the barycentric coordinates (red dots) were calculated. The bar indicates the long axis of the radius.

The 3D bone morphology analysis employed in the present study involved the creation of a 3D model, the construction of a coordinate system for the model, and an analysis of the model using reference points and the shape of the joint surface. Computer analyzing software (Zed-Trauma distal radius stage, LEXI Co., Ltd., Tokyo, Japan, and BoneSimulater, Orthree, Osaka, Japan) was used to analyze the 3D bone model of the distal radius as described by Yoshii et al. [2,9,12]. The DICOM format was employed in the data analysis. After importing image data into the software, the radius bone was segmented according to the CT values. A surface construction algorithm was used to construct a 3D surface model of the distal radius and create a 3D bone model of the radius. The standard triangulated language (STL) data of the 3D model were used for the further analysis. Using data measurement mode in the BoneSimulator, the coordinate system was defined based on 3D data of the distal radius. The long axis of the radius was automatically calculated as follows. The software detected the proximal-to-distal center curve of the radius shaft by analyzing cross-sections at various locations. It then calculated the central point at each level from surface data on the radial diaphysis. An approximate straight line based on the central point was defined as the long axis. The long axis of the radius was defined as the *y*-axis (positive: the proximal direction; negative: the distal direction). The *z*-axis (positive: the radial direction; negative: the ulnar direction) was parallel to the orthogonal projection of the line that originated at the base on the sigmoid notch of the distal radius and continued to the radial styloid process on the plane perpendicular to the *y*-axis. The *x*-axis (positive: the palmar direction; negative: the dorsal direction) was defined as perpendicular to the yz plane. The yz, xy, and xz planes were defined as the coronal, sagittal, and axial planes, respectively. The origin of the coordinate axes was defined as the intersection of the joint surface and the long axis of the radius. The following three reference points were marked on the 3D image: (1) the radial styloid process; (2) sigmoid notch volar edge; and (3) sigmoid notch dorsal edge (Figure 1). The 3D coordinates of each reference point were evaluated using 3D images. In addition, the articular surface area and the position of barycentric coordinates were calculated from the surface shape of the distal radius joint (Figure 2). The articular surface of the 3D model was extracted for identification with the bony prominence of the distal radius, and the articular surface was extracted. The articular surface area and barycentric point were calculated using the preinstalled function in the software.

**Figure 3:**
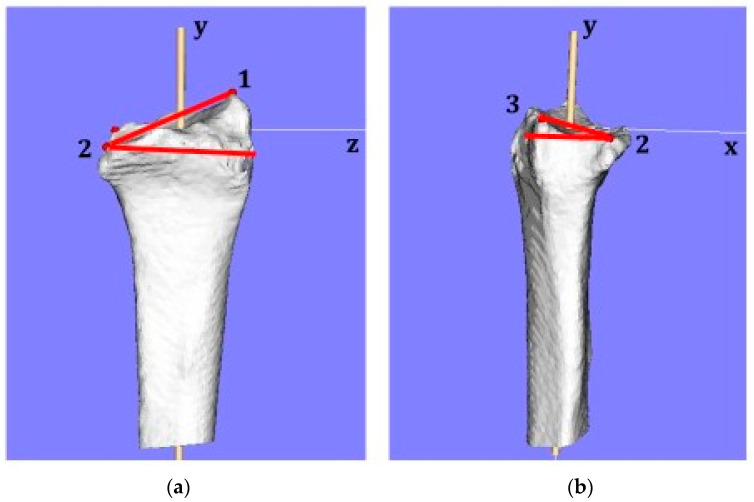
Measurements of 3DRI and 3DPT. (**a**) 3DRI was defined as the angle between the line connecting reference point (1) to reference point (2) and the line perpendicular to the longitudinal axis of the radius. (**b**) 3DPT was defined as the angle between the line connecting reference point (2) to the reference point (3) and the line perpendicular to the longitudinal axis of the radius. Red lines show the angles for the 3DRI and 3DPT.

The 3D measurement indices corresponding to the X-ray measurement were measured. The angle between the line connecting reference point (2) to reference point (3) and the line perpendicular to the longitudinal axis of the radius were measured as the palmar tilt (PT) on a 3D image in the sagittal view. The angle between the line from reference point (1) to reference point (2) and the line perpendicular to the longitudinal axis of the radius were measured as the radial inclination (RI) on a 3D image in the coronal view (Figure 3).

### 2.2. Statistical Analysis

Results are expressed as the mean ± standard deviation. The average positions for the three reference points relative to the origin were analyzed for each reference point. The Shapiro-Wilk test was used to test the normality of datasets. Differences in the parameters for each reference point were compared between males and females. In addition, joint surface areas were compared between males and females. Welch’s *t*-test was used for comparisons between male and female measurements. *p* values less than 0.05 were considered to be significant. All analyses were performed using BellCurve for Excel version 2.12 (SSRI Co., Tokyo, Japan).

## 3. Results

**Figure 4:**
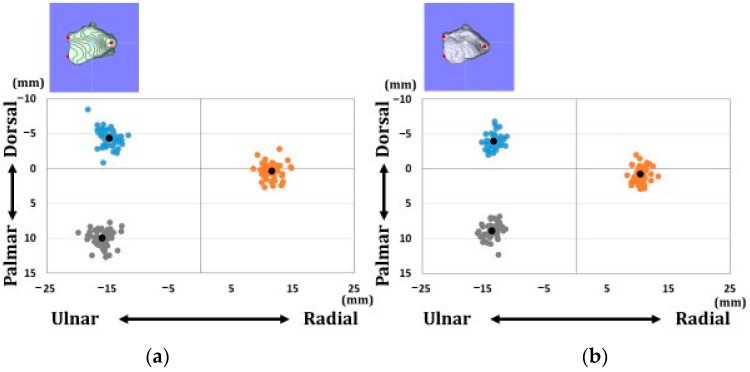
Coordinates of three reference points in the axial plane. (**a**) Results of coordinates for males. (**b**) Results of coordinates for females. Orange dots indicate the radial styloid process: reference point (1). Gray dots indicate the sigmoid notch volar edge: reference point (2). Blue dots indicate the sigmoid notch dorsal edge: reference point (3). Black dots indicate average positions for each reference point.

**Figure 5:**
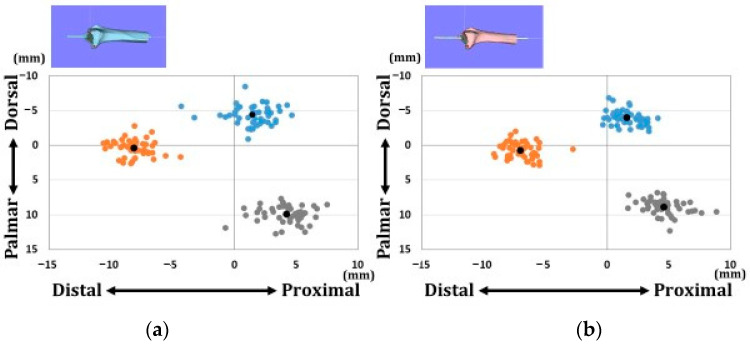
Coordinates of three reference points in the sagittal plane. (**a**) Results of coordinates for males. (**b**) Results of coordinates for females. Orange dots indicate the radial styloid process: reference point (1). Gray dots indicate the sigmoid notch volar edge: reference point (2). Blue dots indicate the sigmoid notch dorsal edge: reference point (3). Black dots indicate average positions for each reference point.

Figure 4 shows the position of each reference point in the axial direction. Figure 5 shows the position of each reference point in the sagittal direction. Variations in each reference point were larger in the sagittal direction than in the axial direction. The transverse diameter of the radius was larger in males than in females. The positions of each reference point from the origin were located at (1) 14.2 ± 1.3/12.7 ± 1.1 mm for the distal–palmar–radial position, (2) 19.3 ± 1.3/16.9 ± 1.3 mm for the proximal–palmar–ulnar position, (3) 15.6 ± 1.4/14.1 ± 0.9 mm for the proximal–dorsal–ulnar position, and (barycenter) 4.1 ± 0.7/3.7 ± 0.7 mm for the proximal–volar–ulnar position for males and females, respectively. Significant differences were observed in all reference points between males and females (*p* < 0.01).

**Figure 6:**
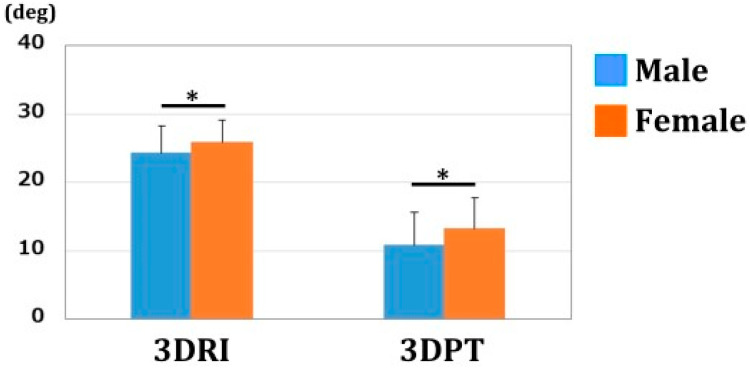
3DRI and 3DPT. Both 3DRI and 3DPT were significantly larger in females than in males (*: *p* < 0.01).

**Figure 7:**
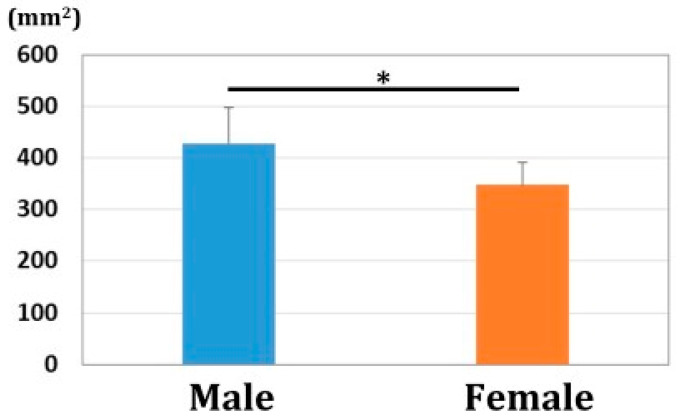
The area of the radius articular surface. The area was significantly larger in males than in females (*: *p* < 0.01).

The values of 3DRI and 3DPT were 24.2 ± 4.0/25.7 ± 3.1° and 10.9 ± 5.1/13.2 ± 4.4° for males and females, respectively. (Figure 6) The areas of the radius articular surface were 429.0 ± 67.9/347.6 ± 44.6 mm^2^ for males and females, respectively. (Figure 7) Significant differences were observed in 3DRI, 3DPT, and the articular surface area between males and females (*p* < 0.01).

## 4. Discussion

The present study attempted to define the standard positional relationships of reference points. To date, X-ray measurements have been used as a measurement index of the wrist joint morphology. RI, PT, and ulnar variance (UV) are regarded as common metrics [13]. On the other hand, measurement errors have been reported for these indicators. We previously evaluated the inter-rater reliability of X-ray measurements and found higher reliability for UV but only moderate inter-rater reliabilities for RI and PT [2].

In a previous study based on simple X-ray measurements of healthy subjects, the average values of RI and volar tilt (VT) were 20–26.6 and 7.9–14.5°, respectively [13,14,15]. The 3DRI and 3DPT measured in the present study were similar to these values. Limited information is currently available on sex differences in these values. In a study that conducted wrist morphology measurements on Indonesians, RI was larger in males than in females, whereas PT was larger in females than in males [16]. Furthermore, 3DRI and 3DPT were both larger in females, indicating differences in RI and PT that are dependent on country and race. In addition, since this measurement is based on a three-dimensional reference point, differences that depend on the measurement method may exist. Recent anatomical studies on Caucasian cadavers revealed a significant angular difference between the lateral and intermediate columns as well as between males and females [17].

In the present study, the long axis of the radius was calculated from the central point at each level from 3D surface data on the radial diaphysis. The optimal location for measuring the longitudinal axis of the radius was previously suggested to be between 28.8 and 53.3 mm from the joint surface [18]. The definition of the long axis of the radius affects the measurement result of each parameter, particularly RI and PT. The findings of morphological research on the distal radius using plain radiographs may have a measurement bias because the definition of the long axis of the radius requires the measurement of each parameter. The present results have the potential to provide true morphological realignment parameters for the distal radius to orthopedic surgeons treating distal radius fractures. We consider accurate realignment parameters to be necessary for anatomical reduction, particularly in cases with bilateral fracture or a history of malunion on the contralateral side.

Surgery for distal radius fractures requires anatomical realignment [19]. Although radiological parameters, such as RI, VT, and UV, of the contralateral side are generally used as indicators of anatomical reduction, the inter-rater reliability of these parameters is not high [20,21]. Furthermore, plain radiographs may yield inconsistent measurements depending on the incidence angle of X-rays and limb position because the articular surface of the distal radius is a complex 3D structure. Suojärvi et al. demonstrated that a computer-aided CT analysis of radiographic parameters was more reliable than the interpretation of radiographs by a physician [21]. Regarding the reliability of 3D parameters, a supplementary evaluation of inter-rater reliability in the data of this case by two orthopedic fellows (5 years of experience) showed that the interclass correlation coefficients of 3DPT and 3DRI were higher than those of PT and RI in X-rays, and measurement errors were significantly smaller in 3D data evaluations. (Figure 1) The inter-rater reliability of X-ray measurements of the distal radius, which are used to evaluate the complex three-dimensional structure of the articular surface in two dimensions, is not high [22]. The present results suggest that measurement errors were fewer in 3D evaluations than in traditional X-ray evaluations because reference points for the measurement of RI and PT were clearly defined from 3D coordinates in this study.

The 3D reference points of the distal radius validated in the present study may be useful for more accurate preoperative planning as an indicator of anatomical reductions in distal radius fractures; however, further verification by CT is required. The present results may be useful as parameters for anatomical reduction, particularly in cases of bilateral fracture or a history of contralateral fracture. Furthermore, preoperative planning using 3D data has the advantage of enabling planning for optimal plate placement, screw insertion directions, and screw lengths after restoration, which will improve outcomes after surgery [9].

There are several limitations that need to be addressed. The measurement of 3D reference points and the barycenter of the articular surface requires CT scans. Although CT has clear advantages in terms of excellent bone–soft tissue contrast and no geometrical distortion, its acquisition exposes patients to radiation; therefore, care is needed to reduce radiation exposure. In a previous study [23], bone morphology was evaluated with high accuracy even when the radiation exposure dose was 1/30 the level of a standard CT scan. This may be one solution to radiation exposure. Furthermore, we did not compare morphological differences between each generation. In the elderly, age-related articular changes, such as osteoarthritis, may affect the standard values for each parameter of the distal radius. Nevertheless, the risk of osteoarthritic changes is lower than that in the knee and hip joints because the wrist is a non-weight bearing joint.

Moreover, we did not compare differences in laterality because we only used data on the unilateral and unaffected wrist from patients with a wrist damaged by trauma.

## 5. Conclusions

The present results suggest sex differences in the 3D morphological parameters of the distal radius in Japanese individuals. The reference points and measured values presented in this study serve as criteria for identifying the dislocation direction and reduction conditions for distal radius fractures in 3D images. The results in this study clearly showed the standard 3D positional relationship of each reference point. Clinically, this may help for the reduction of distal radius fractures, especially when the opposite side is deformed by previous trauma or other pathological conditions. In addition, it was suggested that the ideal reduction shape may be different by gender. This may need to be considered during the fracture reduction.

## Data Availability

The datasets used and/or analyzed during the present study are available from the corresponding author upon reasonable request.
